# 1,3-Bis(carboxy­meth­yl)imidazolium triiodide 1-carboxyl­atomethyl-3-carboxy­methyl­imidazolium

**DOI:** 10.1107/S1600536809025239

**Published:** 2009-07-04

**Authors:** Jinling Miao, Chunhua Hu, Haiyan Chen, Guizhen Yuan, Yong Nie

**Affiliations:** a School of Chemistry and Chemical Engineering, University of Jinan, Jinan 250022, People’s Republic of China; b Department of Chemistry, New York University, 100 Washington Square East, New York, NY 10003-6688, USA

## Abstract

In the title compound, C_7_H_9_N_2_O_4_^+^·I_3_^−^·C_7_H_8_N_2_O_4_, the two imidazolium units are hydrogen bonded through the carboxyl groups. The units are further linked *via* inter­molecular O—H⋯O hydrogen bonding, resulting in a one-dimensional ladder-type structure. As a result, the two carb­oxy groups of each imidazolium unit adopt a *cis* configuration with respect to the imidazolium ring.

## Related literature

For the preparation of 1,3-bis­(carboxy­meth­yl)imidazole, see: Kratochvíl *et al.* (1988 ▶); Fei *et al.* (2004 ▶); Barczynski *et al.* (2008 ▶). For its structure, see: Kratochvíl *et al.* (1988 ▶).
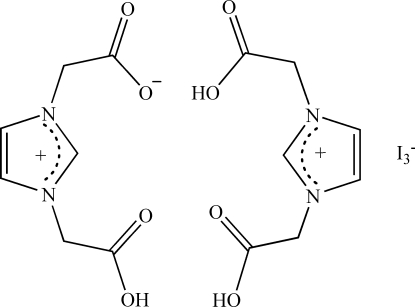


## Experimental

### 

#### Crystal data

C_7_H_9_N_2_O_4_^+^·I_3_^−^·C_7_H_8_N_2_O_4_
*M* *_r_* = 750.02
Monoclinic, 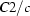

*a* = 22.260 (3) Å
*b* = 10.1973 (17) Å
*c* = 10.1077 (17) Å
β = 92.209 (2)°
*V* = 2292.7 (6) Å^3^
*Z* = 4
Mo *K*α radiation
μ = 4.14 mm^−1^
*T* = 298 K
0.49 × 0.44 × 0.40 mm

#### Data collection

Bruker SMART 1000 CCD area-detector diffractometer
Absorption correction: multi-scan (*SADABS*; Bruker, 2001 ▶) *T*_min_ = 0.237, *T*_max_ = 0.289 (expected range = 0.157–0.191)
6257 measured reflections
2248 independent reflections
1702 reflections with *I* > 2σ(*I*)
*R*_int_ = 0.031

#### Refinement

*R*[*F* ^2^ > 2σ(*F* ^2^)] = 0.031
*wR*(*F* ^2^) = 0.112
*S* = 1.03
2248 reflections
140 parameters
H atoms treated by a mixture of independent and constrained refinement
Δρ_max_ = 0.84 e Å^−3^
Δρ_min_ = −0.99 e Å^−3^

### 

Data collection: *SMART* (Bruker, 2001 ▶); cell refinement: *SAINT* (Bruker, 2001 ▶); data reduction: *SAINT*; program(s) used to solve structure: *SHELXS97* (Sheldrick, 2008 ▶); program(s) used to refine structure: *SHELXL97* (Sheldrick, 2008 ▶); molecular graphics: *SHELXTL* (Sheldrick, 2008 ▶); software used to prepare material for publication: *SHELXTL*.

## Supplementary Material

Crystal structure: contains datablocks I, global. DOI: 10.1107/S1600536809025239/bt2982sup1.cif

Structure factors: contains datablocks I. DOI: 10.1107/S1600536809025239/bt2982Isup2.hkl

Additional supplementary materials:  crystallographic information; 3D view; checkCIF report

## Figures and Tables

**Table 1:** Hydrogen-bond geometry (Å, °)

*D*—H⋯*A*	*D*—H	H⋯*A*	*D*⋯*A*	*D*—H⋯*A*
O3—H3*O*⋯O1^i^	0.81 (8)	1.80 (8)	2.591 (6)	166 (9)
O2—H2*O*⋯O2^ii^	1.224 (4)	1.224 (4)	2.449 (6)	179 (9)

Symmetry codes: (i) 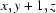; (ii) 
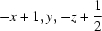
.

**Fig. 1.**
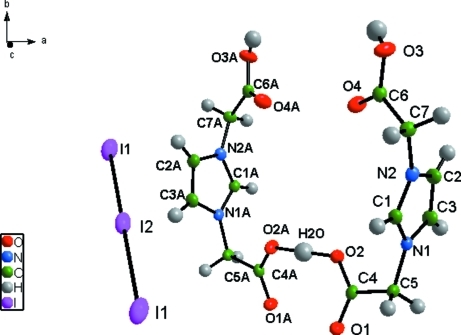
The molecular structure, with atom labels and 25% probability thermal ellipsoids.

**Fig. 2.**
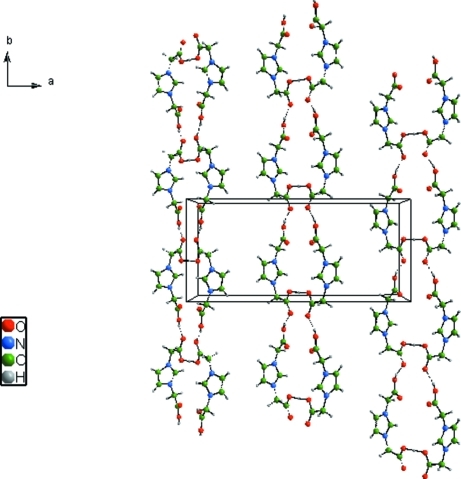
The crystal packing diagram viewed along the c axis (only one layer shown), showing the hydrogen bonds as dotted lines; iodine atoms have been omitted for clarity

## supplementary crystallographic information

### Comment

1,3-bis(carboxymethyl)imidazole was first prepared by the condensation reaction of formaldehyde, glyoxal and glycine (Kratochvĺ *et al.*, 1988). Recently its synthesis by the reaction of alkyl haloacetate with imidazole has been reported (Fei *et al.*, 2004; Barczynski *et al.*, 2008). We have found that the reaction of imidazole with chloroacetic acid in the presence of NaOH as a base produces colorless 1,3-bis(carboxymethyl)imidazole, while the same reaction with iodoacetic acid affords the red title compound.

As shown in Fig. 1, two imidazolium units are hydrogen bonded through the carboxy groups. The presence of an I_3_^-^ anion accounts for the neutral nature of the whole structure. The bond lengths of C4—O1 and C4—O2 are 1.231 (6), 1.259 (6) Å (table 1), respectively, which are between those for a C—O single bond and a C═O double bond. The C—N bond lengths on the rings are found to be within 1.316 (6)–1.384 (6) Å (Table 1), which are between those for a C—N single bond and a C═N double bond, suggesting charge delocalization on the planar imidazolium rings. The two imidazolium units are extended by intermolecular hydrogen bonding (O3-H3O—O1i, [i = x, y+1, z], 2.591 (6) Å) to generate a one-dimensional ladder-type structure along the *c* axis (Fig. 2). As a result of the hydrogen bonding, the two carboxy groups of each imidazolium unit adopt a *cis* configuration, while in the structure of 1,3-bis(carboxymethyl)imidazole (Kratochvĺ *et al.*, 1988) a *trans* configuration has been found.

### Experimental

To a solution of iodoacetic acid (9.314 g, 0.05 mol) in distilled water (25 ml), an aqueous solution (25 ml) of NaOH (2.020 g, 0.05 mol) was added, and followed by the addition of imidazole (2.020 g, 0.03 mol). The resulting colorless solution was heated to reflux during which the color gradually changed to yellow. The pH was adjusted using saturated NaOH solution once per 20 min., keeping in the range of 8–9, till no obvious change observed. The mixture was further refluxed for 30 min. and cooled, acidified with hydrochloric acid till pH 2–3, to give an orange-red solution. After 5 days, deep red crystals (yield 11.5% based on iodoacetic acid) were formed over evaporation. IR (KBr): *v* = 3437, 3117, 1720, 1665, 1350, 1239, 890 cm ^-1^.

### Refinement

H2O and H3O were located on the difference Fourier map. All other H-atoms were positioned geometrically and refined using a riding model with d(C—H) = 0.93 Å, *U*_iso_=1.2*U*_eq_ (C) for aromatic, 0.97 Å, *U*_iso_ = 1.2*U*_eq_ (C) for CH_2_ atoms.

### Crystal data

**Table:**

C_7_H_9_N_2_O_4_^+^·I_3_^−^·C_7_H_8_N_2_O_4_	*F*(000) = 1408
*M**_r_* = 750.02	*D*_x_ = 2.173 Mg m^−^^3^
Monoclinic, *C*2/*c*	Mo *K*α radiation, λ = 0.71073 Å
*a* = 22.260 (3) Å	Cell parameters from 2974 reflections
*b* = 10.1973 (17) Å	θ = 3.0–27.2°
*c* = 10.1077 (17) Å	µ = 4.14 mm^−^^1^
β = 92.209 (2)°	*T* = 298 K
*V* = 2292.7 (6) Å^3^	Plate, red
*Z* = 4	0.49 × 0.44 × 0.40 mm

### Data collection

**Table:**

Bruker SMART 1000 CCD area-detector diffractometer	2248 independent reflections
Radiation source: fine-focus sealed tube	1702 reflections with *I* > 2σ(*I*)
graphite	*R*_int_ = 0.031
φ and ω scans	θ_max_ = 26.0°, θ_min_ = 1.8°
Absorption correction: multi-scan (*SADABS*; Bruker, 2001)	*h* = −18→27
*T*_min_ = 0.237, *T*_max_ = 0.289	*k* = −11→12
6257 measured reflections	*l* = −9→12

### Refinement

**Table:**

Refinement on *F*^2^	Secondary atom site location: difference Fourier map
Least-squares matrix: full	Hydrogen site location: inferred from neighbouring sites
*R*[*F*^2^ > 2σ(*F*^2^)] = 0.031	H atoms treated by a mixture of independent and constrained refinement
*wR*(*F*^2^) = 0.112	*w* = 1/[σ^2^(*F*_o_^2^) + (0.0655*P*)^2^ + 3.4411*P*] where *P* = (*F*_o_^2^ + 2*F*_c_^2^)/3
*S* = 1.03	(Δ/σ)_max_ < 0.001
2248 reflections	Δρ_max_ = 0.84 e Å^−^^3^
140 parameters	Δρ_min_ = −0.98 e Å^−^^3^
0 restraints	Extinction correction: *SHELXL97* (Sheldrick, 2008), Fc^*^=kFc[1+0.001xFc^2^λ^3^/sin(2θ)]^-1/4^
Primary atom site location: structure-invariant direct methods	Extinction coefficient: 0.0032 (2)

### Fractional atomic coordinates and isotropic or equivalent isotropic displacement parameters (Å^2^)

**Table:**

	*x*	*y*	*z*	*U*_iso_*/*U*_eq_	
O1	0.46232 (19)	−0.1010 (4)	0.1047 (4)	0.0471 (10)	
O2	0.45813 (18)	0.1069 (4)	0.1678 (4)	0.0467 (10)	
O3	0.4311 (2)	0.7258 (4)	−0.0724 (5)	0.0608 (13)	
O4	0.4461 (2)	0.5756 (4)	0.0836 (4)	0.0533 (11)	
N1	0.38543 (17)	0.1836 (4)	−0.0301 (4)	0.0331 (9)	
N2	0.38902 (19)	0.3887 (4)	−0.0756 (4)	0.0337 (9)	
C1	0.4130 (2)	0.2736 (5)	−0.0999 (5)	0.0349 (11)	
H1	0.4441	0.2581	−0.1566	0.042*	
C2	0.3446 (2)	0.3728 (5)	0.0145 (6)	0.0415 (12)	
H2	0.3207	0.4385	0.0487	0.050*	
C3	0.3423 (3)	0.2448 (5)	0.0434 (6)	0.0431 (13)	
H3	0.3167	0.2047	0.1016	0.052*	
C4	0.4439 (2)	0.0124 (5)	0.0929 (5)	0.0357 (11)	
C5	0.4008 (2)	0.0437 (5)	−0.0226 (5)	0.0362 (11)	
H5A	0.4189	0.0175	−0.1043	0.043*	
H5B	0.3643	−0.0069	−0.0140	0.043*	
C6	0.4295 (2)	0.6072 (5)	−0.0261 (5)	0.0365 (11)	
C7	0.4056 (3)	0.5151 (5)	−0.1311 (5)	0.0381 (12)	
H7A	0.3706	0.5541	−0.1757	0.046*	
H7B	0.4359	0.5019	−0.1962	0.046*	
I1	0.228024 (19)	0.42974 (5)	0.27861 (4)	0.0609 (2)	
I2	0.2500	0.2500	0.5000	0.0519 (2)	
H2O	0.5000	0.106 (9)	0.2500	0.08 (3)*	
H3O	0.435 (4)	0.780 (7)	−0.014 (9)	0.07 (2)*	

### Atomic displacement parameters (Å^2^)

**Table:**

	*U*^11^	*U*^22^	*U*^33^	*U*^12^	*U*^13^	*U*^23^
O1	0.054 (2)	0.037 (2)	0.049 (2)	0.0015 (17)	−0.0163 (19)	−0.0009 (17)
O2	0.052 (2)	0.044 (2)	0.042 (2)	0.0067 (17)	−0.0205 (18)	−0.0129 (17)
O3	0.094 (4)	0.038 (2)	0.048 (3)	−0.016 (2)	−0.026 (2)	0.009 (2)
O4	0.078 (3)	0.051 (2)	0.030 (2)	−0.0123 (19)	−0.014 (2)	0.0074 (17)
N1	0.030 (2)	0.035 (2)	0.034 (2)	0.0008 (17)	−0.0065 (17)	−0.0041 (18)
N2	0.038 (2)	0.040 (2)	0.023 (2)	−0.0028 (18)	−0.0028 (17)	−0.0004 (17)
C1	0.034 (3)	0.040 (3)	0.031 (3)	0.002 (2)	0.001 (2)	−0.005 (2)
C2	0.036 (3)	0.046 (3)	0.043 (3)	0.003 (2)	0.010 (2)	0.000 (2)
C3	0.038 (3)	0.049 (3)	0.044 (3)	−0.002 (2)	0.012 (2)	0.000 (2)
C4	0.032 (3)	0.042 (3)	0.033 (3)	−0.005 (2)	−0.004 (2)	−0.001 (2)
C5	0.035 (3)	0.035 (3)	0.038 (3)	0.000 (2)	−0.010 (2)	−0.005 (2)
C6	0.036 (3)	0.044 (3)	0.030 (3)	−0.001 (2)	−0.001 (2)	0.006 (2)
C7	0.050 (3)	0.038 (3)	0.025 (2)	−0.003 (2)	−0.004 (2)	0.002 (2)
I1	0.0478 (3)	0.0875 (4)	0.0468 (3)	0.0116 (2)	−0.00472 (19)	−0.0100 (2)
I2	0.0379 (3)	0.0668 (4)	0.0508 (4)	0.0012 (2)	−0.0021 (2)	−0.0218 (3)

### Geometric parameters (Å, °)

**Table:**

O1—C4	1.231 (6)	C1—H1	0.9300
O2—C4	1.259 (6)	C2—C3	1.339 (7)
O2—H2O	1.224 (4)	C2—H2	0.9300
O3—C6	1.297 (6)	C3—H3	0.9300
O3—H3O	0.81 (8)	C4—C5	1.516 (7)
O4—C6	1.199 (7)	C5—H5A	0.9700
N1—C1	1.323 (6)	C5—H5B	0.9700
N1—C3	1.384 (6)	C6—C7	1.500 (7)
N1—C5	1.467 (6)	C7—H7A	0.9700
N2—C1	1.316 (6)	C7—H7B	0.9700
N2—C2	1.380 (6)	I1—I2	2.9192 (6)
N2—C7	1.459 (6)	I2—I1^i^	2.9192 (6)
			
C4—O2—H2O	125 (4)	O1—C4—C5	118.1 (5)
C6—O3—H3O	112 (6)	O2—C4—C5	116.0 (5)
C1—N1—C3	108.5 (4)	N1—C5—C4	112.6 (4)
C1—N1—C5	126.2 (4)	N1—C5—H5A	109.1
C3—N1—C5	125.1 (4)	C4—C5—H5A	109.1
C1—N2—C2	108.9 (4)	N1—C5—H5B	109.1
C1—N2—C7	127.3 (4)	C4—C5—H5B	109.1
C2—N2—C7	123.7 (4)	H5A—C5—H5B	107.8
N2—C1—N1	108.7 (4)	O4—C6—O3	124.9 (5)
N2—C1—H1	125.7	O4—C6—C7	125.0 (5)
N1—C1—H1	125.7	O3—C6—C7	110.0 (5)
C3—C2—N2	107.0 (4)	N2—C7—C6	111.7 (4)
C3—C2—H2	126.5	N2—C7—H7A	109.3
N2—C2—H2	126.5	C6—C7—H7A	109.3
C2—C3—N1	106.9 (5)	N2—C7—H7B	109.3
C2—C3—H3	126.6	C6—C7—H7B	109.3
N1—C3—H3	126.6	H7A—C7—H7B	107.9
O1—C4—O2	125.8 (5)	I1^i^—I2—I1	180.0

Symmetry codes: (i) −*x*+1/2, −*y*+1/2, −*z*+1.

### Hydrogen-bond geometry (Å, °)

**Table:**

*D*—H···*A*	*D*—H	H···*A*	*D*···*A*	*D*—H···*A*
O3—H3O···O1^ii^	0.81 (8)	1.80 (8)	2.591 (6)	166 (9)
O2—H2O···O2^iii^	1.22 (1)	1.22 (1)	2.449 (6)	179 (9)

Symmetry codes: (ii) *x*, *y*+1, *z*; (iii) −*x*+1, *y*, −*z*+1/2.
